# Gastrointestinal Basidiobolomycosis: A Rare Fungal Disease Mimicking Colon Cancer

**DOI:** 10.7759/cureus.96232

**Published:** 2025-11-06

**Authors:** Ali Al Hassani, Aqeel Saleem, Aya Shubbar, Zaid Al Hassani, Muhammed Rashid

**Affiliations:** 1 Infectious Disease, Tawam Hospital, Al Ain, ARE; 2 Infectious Disease, Sheikh Tahnoon Medical City, Al Ain, ARE; 3 Internal Medicine, Sheikh Tahnoon Medical City, Al Ain, ARE; 4 General Medicine, Sheikh Tahnoon Medical City, Al Ain, ARE

**Keywords:** antifungal therapy, basidiobolomycosis, colonic neoplasms, eosinophilic colitis, splendore–hoeppli phenomenon

## Abstract

Gastrointestinal basidiobolomycosis (GIB) is an uncommon fungal infection caused by *Basidiobolus ranarum*, an environmental saprophyte found in soil, decaying vegetation, and the gastrointestinal tracts of reptiles and amphibians. It primarily affects immunocompetent individuals and can closely mimic colonic malignancy or inflammatory bowel disease, often leading to diagnostic delays. We describe the case of a 52-year-old immunocompetent South Asian male who presented with right lower quadrant pain, diarrhea, and coffee-ground vomiting. Colonoscopy with superficial biopsies suggested eosinophilic colitis, while CT raised suspicion for right-sided colonic carcinoma. Due to progressive symptoms and risk of obstruction, surgical resection was performed. Histopathology revealed broad, pauciseptate fungal hyphae surrounded by the Splendore-Hoeppli phenomenon, confirming GIB. The patient is currently undergoing a six-month course of oral itraconazole and, at the six-week follow-up, showed marked improvement, including weight gain and resolution of symptoms.

GIB should be considered in patients from endemic areas who present with abdominal masses, eosinophilia, and a poor response to immunosuppressive therapy. Early diagnosis through deep tissue sampling enables timely initiation of antifungal treatment and may help prevent unnecessary surgical intervention.

## Introduction

Basidiobolomycosis is a rare fungal infection caused by *Basidiobolus ranarum*, a saprophytic filamentous fungus belonging to the order *Entomophthorales*. It naturally inhabits soil, decaying organic matter, and the gastrointestinal tracts of cold-blooded vertebrates, including amphibians and reptiles [1]. While subcutaneous involvement has been recognized for decades, gastrointestinal basidiobolomycosis (GIB) remains rare. However, an increasing number of cases have been reported over the past two decades, particularly from tropical and subtropical regions, including Saudi Arabia and several African countries [2,3]. GIB typically affects immunocompetent individuals, with a predominance in middle-aged men, and most often involves the cecum, ascending colon, and ileocecal region [2,3].

Clinically and radiologically, the disease can closely resemble malignant neoplasms, lymphoma, or inflammatory bowel disease, which leads to frequent misdiagnosis and delays in appropriate treatment [2]. The hallmark histopathologic finding is a dense eosinophilic granulomatous inflammation containing broad, thin-walled, sparsely septate fungal hyphae surrounded by intensely eosinophilic material, a feature known as the Splendore-Hoeppli phenomenon [1,3]. Due to its rarity, nonspecific presentation, and the difficulty of obtaining sufficiently deep diagnostic tissue samples, many patients undergo unnecessary bowel resections before the correct diagnosis is reached [2]. We present a case of GIB in an immunocompetent adult, initially misdiagnosed as eosinophilic colitis and suspected right-sided colonic carcinoma, which was ultimately confirmed through postoperative histopathological examination.

## Case presentation

A 52-year-old South Asian male with no known comorbidities initially presented with a three-day history of watery diarrhea, coffee-ground emesis, and right lower quadrant abdominal pain. The abdominal pain was constant, cramping in nature, and rated at 7/10 in intensity. He denied fever, chills, night sweats, recent travel, or any prior gastrointestinal disorders, and reported no weight loss at the time of presentation. He was not on any regular medications, had no known drug allergies, and denied smoking, alcohol consumption, or recreational drug use. Over the following weeks, his symptoms evolved to include persistent right iliac fossa pain, progressive constipation, and significant unintentional weight loss.

On admission, the patient was afebrile, hemodynamically stable, and in no acute distress. Abdominal examination revealed mild right iliac fossa tenderness without guarding, rebound, or palpable masses; there was no hepatosplenomegaly. No peripheral stigmata of chronic liver disease or cutaneous lesions were observed. Laboratory tests showed leukocytosis with marked eosinophilia, thrombocytosis, elevated C-reactive protein, and acute kidney injury; liver enzymes and amylase/lipase were normal (Table 1).

**Table 1:** Laboratory investigations on admission AKI: acute kidney injury

Parameter	Result	Reference range	Interpretation
White blood cell count	19.7 × 10⁹/L	4.0–11.0 × 10⁹/L	Elevated
Eosinophils (%)	19.70%	0–6%	Markedly elevated
Absolute eosinophil count (AEC)	3.88 × 10⁹/L (≈3,880/µL)	0.02–0.50 × 10⁹/L (20–500/µL)	Marked eosinophilia
Platelet count	575 × 10⁹/L	150–450 × 10⁹/L	Elevated
C-reactive protein	77.3 mg/L	<5 mg/L	Elevated
Serum creatinine	185 µmol/L	60–110 µmol/L	Elevated (AKI)
Liver function tests	Within normal limits	—	—
Amylase/lipase	Within normal limits	—	—

**Figure 1:**
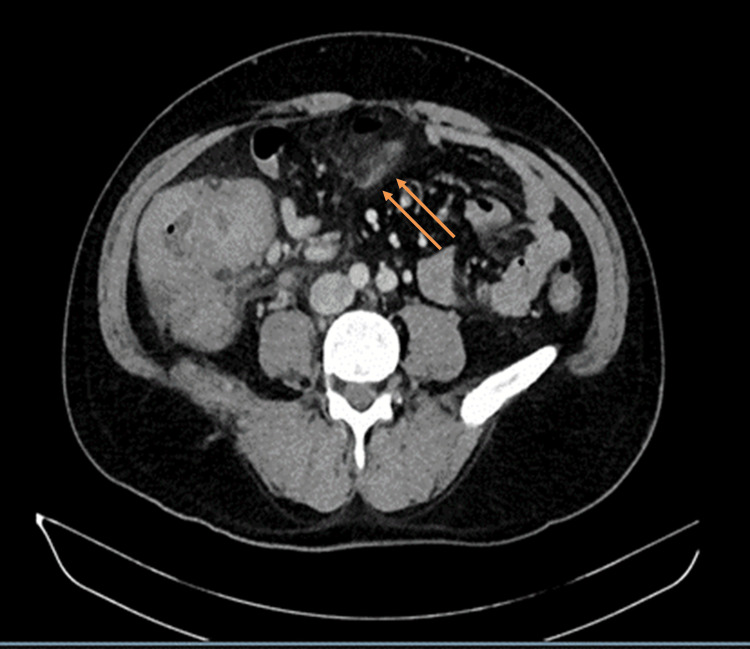
Contrast-enhanced CT scan of the abdomen showing right-sided colonic mass suggestive of malignancy Axial CT image demonstrating circumferential thickening of the cecum and ascending colon (white arrow) with luminal narrowing, mesocolic fat stranding, and multiple small regional lymph nodes (short-axis <14 mm), findings most suggestive of a right-sided colonic malignancy CT: computed tomography

An abdominal ultrasound performed on the day of admission demonstrated bowel wall thickening in the right lower quadrant with minimal surrounding free fluid. A contrast-enhanced CT scan of the abdomen obtained on the same day (Figure 1) showed circumferential thickening of the cecum and ascending colon with luminal narrowing, associated mesocolic fat stranding, and regional lymphadenopathy (short-axis diameter <14 mm). No free intraperitoneal air was detected, and only minimal pelvic free fluid was noted. The overall radiological impression favored a neoplastic process, most likely right-sided colonic adenocarcinoma (T3N2M0), while colitis and ischemic colitis were considered less likely.

Over the following weeks, the patient developed persistent right iliac fossa pain, progressive constipation, and 13-kg unintentional weight loss. In the second week after admission, he was referred to the Gastroenterology Clinic for further evaluation. Stool testing detected *Helicobacter pylori* antigen, and he completed a 14-day eradication regimen according to institutional protocol; this was deemed an incidental finding unrelated to the colonic pathology. Additionally, the fecal immunochemical test (FIT) was positive.

**Figure 2:**
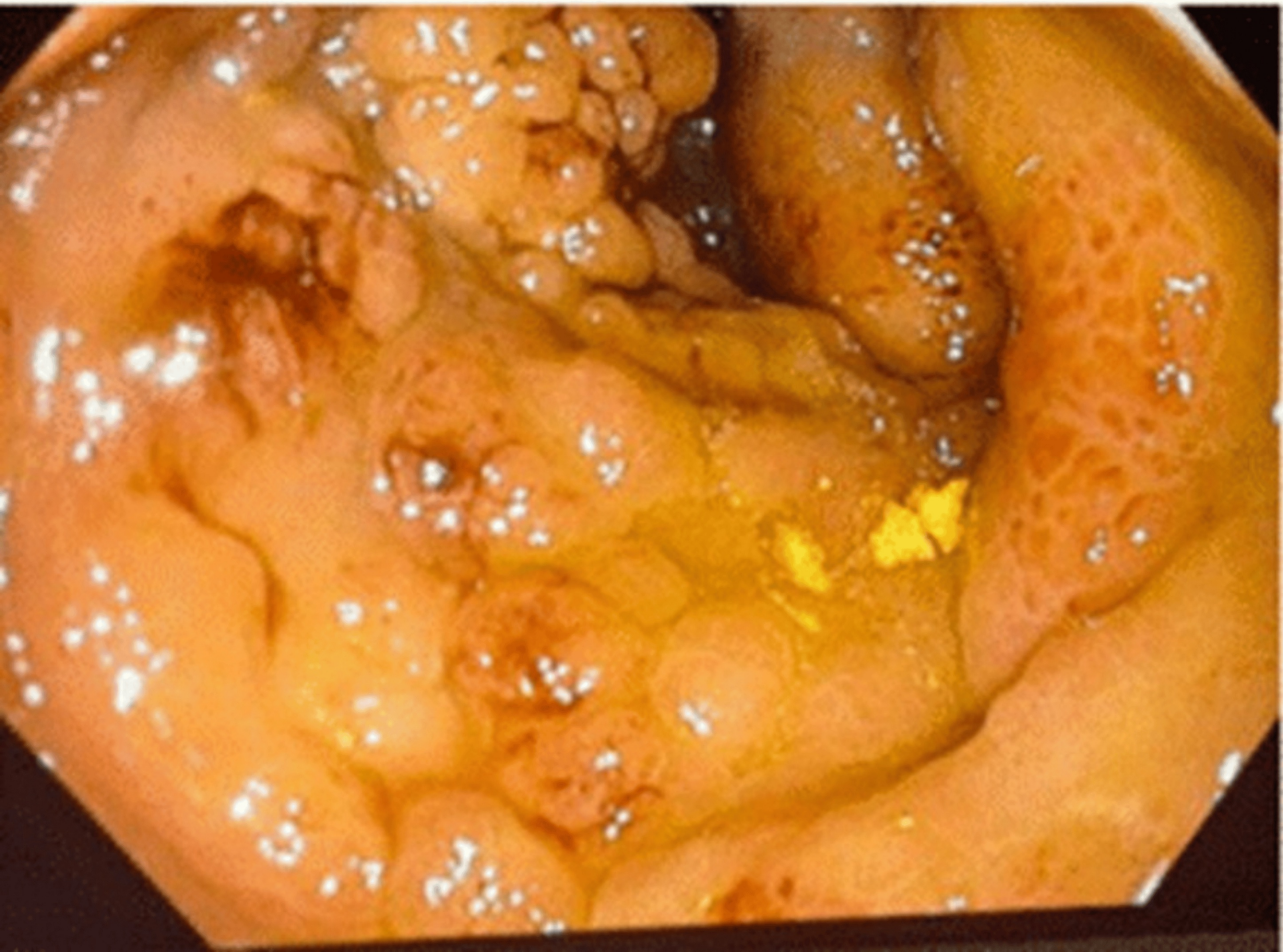
Colonoscopic view of ascending colon mass Endoscopic image showing a large, friable, polypoidal mass occupying the lumen of the ascending colon, with partial obstruction impeding advancement to the cecum. The appearance raised suspicion related to a malignant process

A colonoscopy performed during the fifth week after admission (Figure 2) revealed a large polypoidal mass occupying the ascending colon with partial luminal obstruction, preventing advancement of the scope to the cecum. The lesion appeared malignant, and the differential diagnosis included colonic adenocarcinoma, neuroendocrine tumor, and an inflammatory mass. Multiple biopsies were obtained for histopathological analysis.

Histopathological examination of the colonoscopic biopsies demonstrated dense eosinophilic infiltration (>70 eosinophils/high-power field), eosinophilic cryptitis, and microabscess formation. No dysplasia, malignancy, or fungal elements were identified. The overall features were interpreted as consistent with eosinophilic colitis. Histologic findings are reported as documented in the official pathology report; de-identified photomicrographs could not be obtained under current institutional policies. Based on the initial histological impression of eosinophilic colitis, the patient was commenced on high-dose oral prednisolone (40 mg daily) and 5-aminosalicylic acid (5-ASA). Despite this regimen, he experienced progressive worsening of abdominal pain and anorexia.

**Figure 3:**
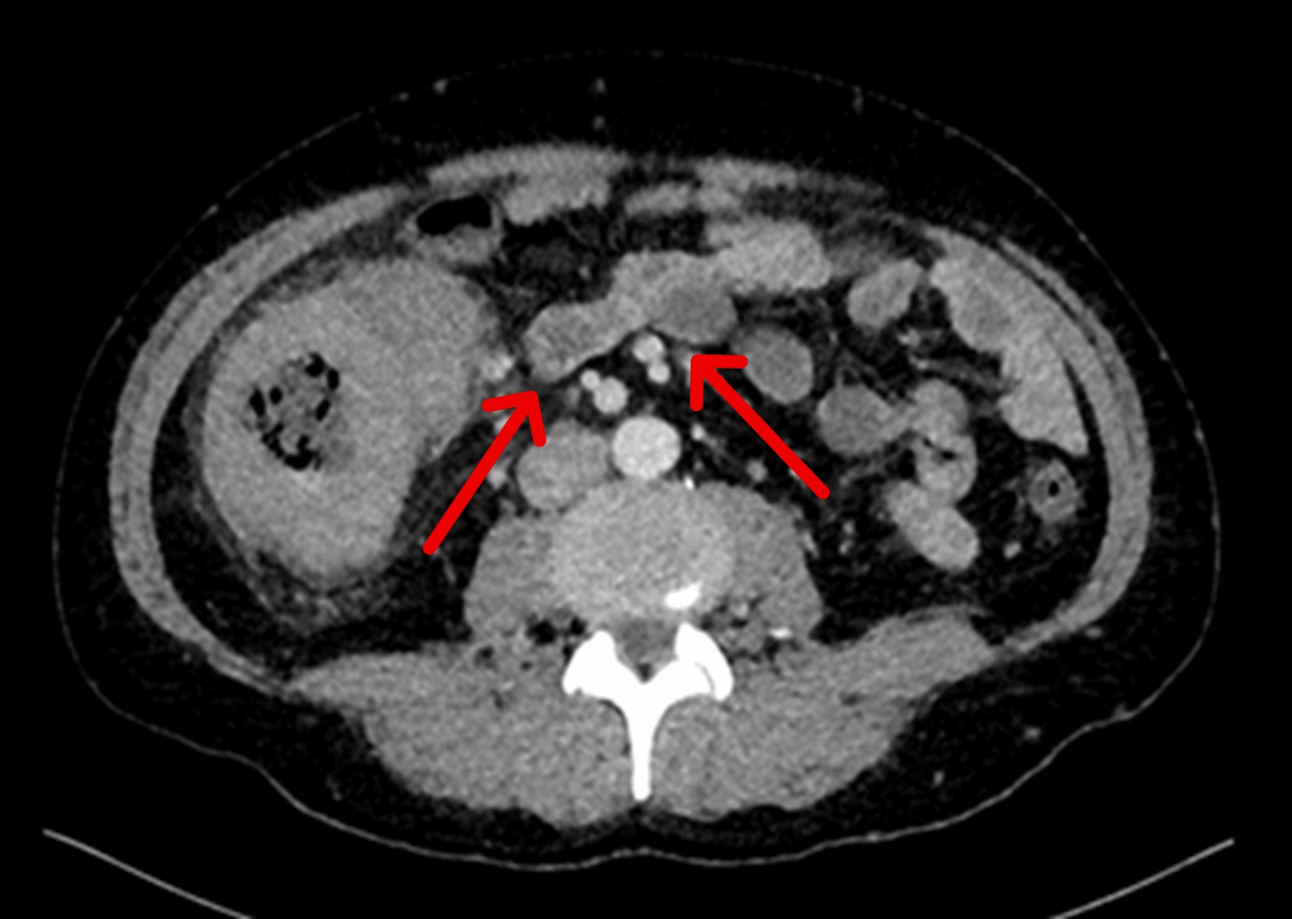
Contrast-enhanced CT scan of the abdomen and pelvis Axial CT image showing diffuse, circumferential wall thickening of the right colon extending to the cecum, ileocecal valve, terminal ileum, and appendix, with pronounced pericolic fat stranding and reactive mesenteric lymphadenopathy. The imaging features were suggestive of a progressive inflammatory or infectious process CT: computed tomography

A repeat contrast-enhanced CT scan of the abdomen and pelvis was performed during week six of illness (Figure 3). It demonstrated progressive circumferential wall thickening involving the entire right colon, cecum, ileocecal valve, terminal ileum, and appendix. There was marked pericolic fat stranding, engorgement of the ileocolic vessels, and multiple reactive mesenteric lymph nodes. No pneumoperitoneum was observed. The radiological differential diagnosis at this stage favored an aggressive infectious or inflammatory process, with neoplastic etiologies such as lymphoma considered less likely. Given the documented disease progression despite immunosuppressive therapy and the increasing risk of bowel obstruction, a decision was made to proceed with surgical intervention.

At six weeks of illness, the patient underwent a laparoscopic right hemicolectomy with primary ileocolic anastomosis. Intraoperatively, a firm, circumferential inflammatory mass was identified involving the right colon and extending to the terminal ileum, with associated mesocolic lymphadenopathy. There was no evidence of peritoneal carcinomatosis, ascites, or distant metastasis. The resected specimen was sent for histopathological evaluation.

Histopathological examination of the resected right hemicolectomy specimen revealed marked granulomatous inflammation composed of epithelioid histiocytes, multinucleated giant cells, and a dense eosinophilic infiltrate. Within the inflammatory tissue, broad, thin-walled, sparsely septate fungal hyphae were identified, surrounded by intensely eosinophilic amorphous material characteristic of the Splendore-Hoeppli phenomenon. Special stains, including periodic acid-Schiff (PAS) and Grocott’s methenamine silver (GMS), highlighted the fungal elements. The findings were diagnostic of GIB.

Following confirmation of the diagnosis, oral itraconazole at a dose of 200 mg twice daily was initiated, with a planned treatment duration of at least six months. Over the subsequent six weeks, the patient demonstrated marked clinical improvement, including resolution of abdominal pain, return of appetite, and progressive weight gain. The clinical course is summarized in Table 2.

**Table 2:** Timeline depicting key clinical events, investigations, management, and outcomes ED: emergency department; RLQ: right lower quadrant; RIF: right iliac fossa; CRP: C-reactive protein; AKI: acute kidney injury; LFTs: liver function tests; IV: intravenous; CT: computed tomography; US: ultrasound; *H. pylori: Helicobacter pylori; *FIT: fecal immunochemical test; 5-ASA: 5-aminosalicylic acid; PAS: periodic acid-Schiff; GMS: Grocott’s methenamine silver

Time point	Event	Key findings and management	Outcome/notes
Day 0 (initial presentation)	ED presentation	3-day history of watery diarrhea, coffee-ground emesis, RLQ pain 7/10; no fever/chills/night sweats/travel; no weight loss	Immunocompetent; no meds/allergies; non-smoker; no alcohol/drugs
Day 0 (admission)	Clinical assessment and labs	Afebrile, stable; mild RIF tenderness, no guarding/rebound; labs: leukocytosis, marked eosinophilia, thrombocytosis, ↑CRP, AKI; LFTs/amylase/lipase normal. IV fluids given for AKI	—
Day 0 (imaging)	Ultrasound and CT abdomen	US: RLQ bowel wall thickening, minimal free fluid. CT: circumferential thickening of cecum/ascending colon with luminal narrowing, fat stranding, small regional nodes; impression favored right-sided colonic malignancy	No free air; minimal pelvic fluid
Weeks 1–2	Symptom evolution	Persistent RIF pain, progressive constipation; total 13-kg unintentional weight loss	—
Week 2 (clinic)	Gastroenterology referral and stool tests	*H. pylori* antigen positive; FIT positive. Completed 14-day *H. pylori* eradication per protocol; finding considered incidental to colonic process	—
Week 5	Colonoscopy with biopsies	Large polypoidal ascending colon mass causing partial obstruction; scope not advanced to cecum. Multiple biopsies obtained	Appearance suspicious for malignancy or inflammatory mass
Week 5 (pathology)	Histology of biopsies	Dense eosinophilic infiltration (>70 eos/HPF), eosinophilic cryptitis, microabscesses; no dysplasia/malignancy/fungal elements → interpreted as eosinophilic colitis	—
Week 5 (treatment)	Empiric medical therapy	Started prednisolone 40 mg daily and 5-ASA for presumed eosinophilic colitis with marked eosinophilia in an endemic context	Worsening abdominal pain and anorexia
Week 6	Repeat CT abdomen/pelvis	Progressive right colon/cecum/ICV/terminal ileum/appendix wall thickening; marked fat stranding; reactive mesenteric nodes; no pneumoperitoneum. MDT decision for surgery due to progression/obstruction risk	Progression despite immunosuppression
Week 6 (operation)	Laparoscopic right hemicolectomy	Intraop: firm circumferential inflammatory mass to the terminal ileum; mesocolic lymphadenopathy; no carcinomatosis/ascites/metastasis. Right hemicolectomy with primary ileocolic anastomosis performed	Specimen sent for histopathology
Post-op week 6 (final pathology)	Histopathology of resection	Granulomatous inflammation with dense eosinophils; broad sparsely septate hyphae with Splendore–Hoeppli; PAS/GMS positive → diagnostic of gastrointestinal basidiobolomycosis	—
Post-op week 6 (therapy)	Antifungal treatment	Started itraconazole 200 mg twice daily; planned for ≥6 months	—
Follow-up week 12	Clinical review	Continued itraconazole. Resolution of abdominal pain, return of appetite, and progressive weight gain	Ongoing clinical improvement

## Discussion

GIB is a rare but increasingly recognized fungal infection caused by *Basidiobolus ranarum*, an environmental saprophyte that resides in soil, decaying vegetation, and the gastrointestinal tracts of reptiles and amphibians [2-4]. Unlike most invasive fungal infections, it often occurs in individuals with no underlying immune compromise [3,5,6]. Men and children are more frequently affected, and the disease has a marked tendency to involve the ileocecal region and ascending colon [3,7]. The infection provokes a chronic pyogranulomatous inflammatory response that is rich in eosinophils [1,3,5]. A distinctive histopathological hallmark is the Splendore-Hoeppli phenomenon, in which broad, sparsely septate fungal hyphae are surrounded by dense eosinophilic material [3,8].

In recent years, cases of GIB have risen in arid and tropical regions, notably in Saudi Arabia, other Middle Eastern countries, and certain parts of the United States [3,9]. Despite this growing number of documented cases, the condition remains underdiagnosed worldwide [2,3,9], largely due to limited clinician awareness, its rarity, and its tendency to mimic more common gastrointestinal diseases, which often results in delayed diagnosis and suboptimal management [5,8].

This report illustrates several of the diagnostic pitfalls that make GIB challenging to recognize. On cross-sectional imaging, the disease may present with concentric bowel wall thickening, pericolic fat stranding, and regional lymphadenopathy, features that are often indistinguishable from those seen in colonic adenocarcinoma or lymphoma [8]. Endoscopic biopsies, if superficial, frequently fail to capture the deeper tissue layers where the fungal elements reside [2,3]. When this happens, the pathology report may show only dense eosinophilic infiltration, leading to alternative diagnoses such as eosinophilic colitis [3,8]. Hence, obtaining deeper biopsies or surgical specimens is essential to confirm the presence of the characteristic fungal hyphae [1-3,5].

Clinicians should have a high index of suspicion for GIB in patients presenting with abdominal pain, altered bowel habits, or rectal bleeding, particularly when these symptoms are associated with an abdominal mass and peripheral eosinophilia in individuals from endemic areas [5,10]. The gold standard for diagnosis is tissue culture, which allows for precise identification of the organism [5,7]. Histopathology can be almost as conclusive when it reveals the classic features of *Basidiobolus ranarum*, including chronic granulomatous inflammation rich in eosinophils and the Splendore-Hoeppli phenomenon [3,5]. Additionally, molecular techniques have been utilized in cases where the diagnosis remains uncertain [3,5]. In 2014, El-Shabrawi et al. reported a method using 18S rRNA gene sequencing to confirm *Basidiobolus ranarum* in tissue samples with equivocal histology, offering a valuable tool for complex cases [4].

From a therapeutic standpoint, itraconazole remains the most widely used antifungal agent and is associated with favorable outcomes, particularly when combined with surgical resection [7]. Voriconazole has been successfully employed as a second-line treatment in patients who do not respond to itraconazole or who cannot tolerate it [6,9]. The importance of timely treatment is underscored by case reports documenting rapid clinical improvement. One such case demonstrated marked symptom resolution and normalization of laboratory parameters, including eosinophil count, within just 10 days of starting itraconazole [10].

Overall, the best results are achieved through a combination of medical and surgical management. However, when the disease is detected early, antifungal therapy alone may be sufficient, sparing the patient from extensive surgical intervention [1]. Close follow-up with clinical assessment and, where appropriate, imaging is important to ensure complete resolution and to detect any signs of recurrence.

## Conclusions

GIB should be suspected in patients from endemic regions who present with a right-sided colonic mass and peripheral eosinophilia, particularly when superficial biopsies are nondiagnostic. In this case, progressive symptoms with impending obstruction and interval radiologic worsening prompted right hemicolectomy, which provided both source control and diagnostic tissue. When patients are clinically stable without obstruction, perforation, or sepsis, and when deep tissue sampling confirms the diagnosis, antifungal therapy alone, most commonly itraconazole, may be sufficient. Early pursuit of adequate-depth sampling with fungal stains and culture, with molecular testing when available, can shorten the time to diagnosis and reduce the likelihood of unnecessary surgery. Surgery is warranted in cases of clinical deterioration or complications. Close clinical follow-up is important, as antifungal therapy often necessitates a prolonged treatment course.
